# Intraocular myofibroblastoma in an infant: a case report

**DOI:** 10.1186/s12886-015-0082-3

**Published:** 2015-08-25

**Authors:** Ning Hua, Jinyong Lin, Xuehan Qian, Nan Wei, Shaozhen Zhao

**Affiliations:** Tianjin Medical University Eye Hospital, Tianjin, China; Tianjin Eye Hospital, Tianjin, China

**Keywords:** Intraocular neoplasm, Myofibroblastoma, Infancy

## Abstract

**Background:**

Myofibroblastoma is a benign tumor composed of spindle cells and bands of hyalinized collagen. Intraocular myofibroblastoma in infancy is rarely encountered.

**Case presentation:**

The present study reports the case of a 4-month-old female baby with intraocular myofibroblastoma. She was suspected as corneal perforation due to the rupture of a corneal neoplasm in the right eye. The anterior segment was also involved according to the Color Doppler ultrasonography. A surgical exploration was performed and the protuberant part of the mass was resected. Conventional HE staining showed numerous spindle-shaped cells with bands of collagen beneath multilayers of well-differentiated corneal epithelia. Immunohistochemical staining demonstrated the tumor cells were strong positive for vimentin and smooth muscle actin, while negative for S-100 protein. The mass was confirmed as myofibroblastoma. After 12 month follow-up, there was no apparent growth of the tumor.

**Conclusions:**

Myofibroblastoma is a very rare type of intraocular neoplasm, which may have complicated manifestation and could be misdiagnosed as dermoid or Peter’s anomaly. Histopathological and immunohistochemical staining is crucial to form a precise diagnosis.

## Background

Myofibroblastoma is a benign mesenchymal neoplasm composed of spindle-shaped cells as well as interspersed bands of hyalinized collagen [1]. Histopathological and immunohistochemical staining is important to differentiate it from other mesenchymal tumors [2–6]. Although the majority involvement of the mass is the breast, extramammary myofibroblastoma was also reported in different organs [2, 3, 6]. However, only one case of orbital and ocular myofibroblastoma was diagnosed for an adult recently [7]. In this study we described a 4-month-old female infant with an intraocular neoplasm in the right eye. By means of Histopathological and immunohistochemical staining, it was confirmed as a rare myofibroblastoma. To the best of our knowledge, this is the first study to show the presence of myofibroblastoma in the right eyeball in infancy.

## Case presentation

**Fig. 1:**
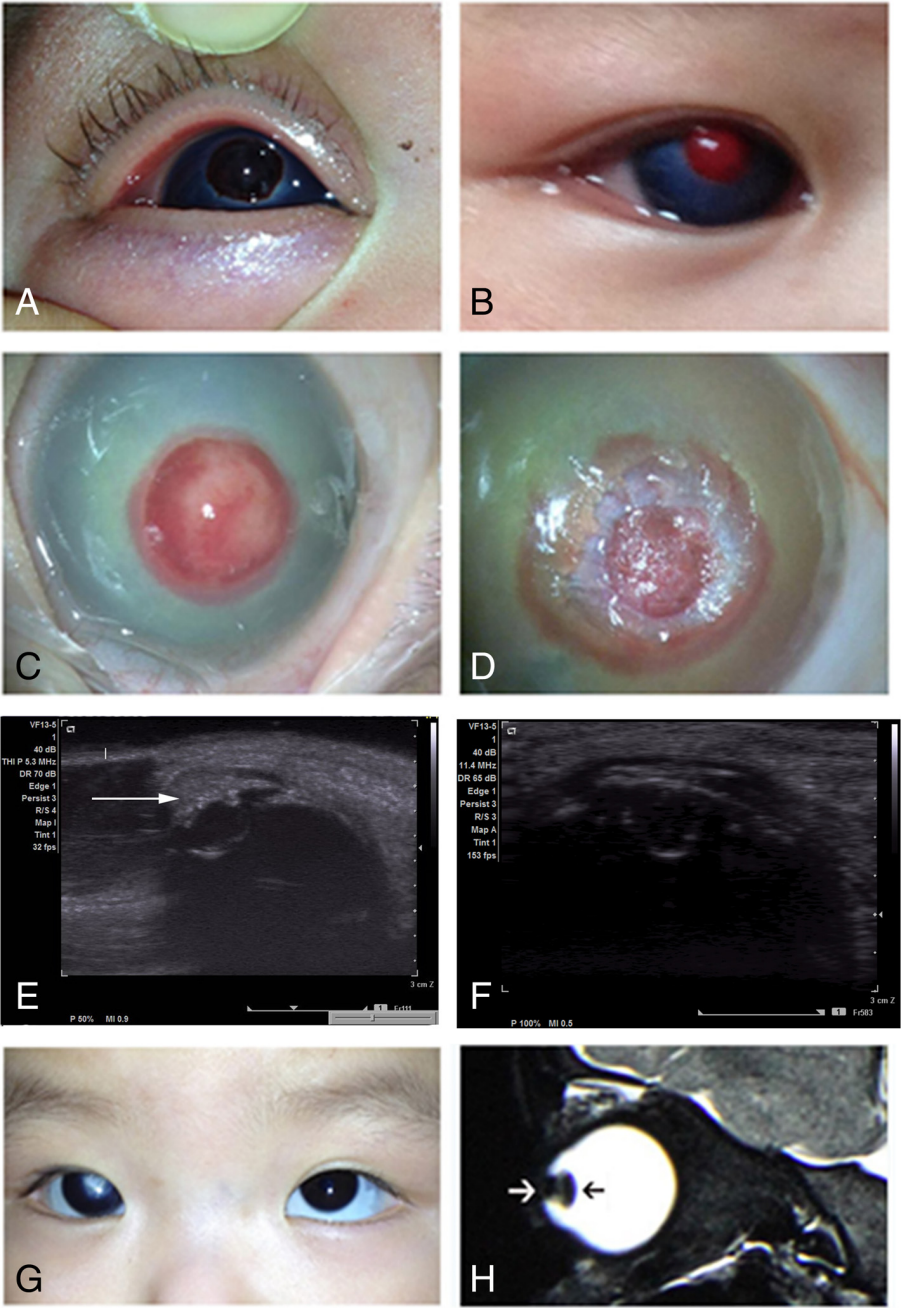
Physical appearance and imaging examination results of the right eye before and after the operation. **a** A black hemispherical neoplasm was observed in the central cornea of the right eye on the day of admission. **b** The hemispherical corneal neoplasm in the right eye turned red on the next day after admission. **c** Preoperative microscopic image (day 4 after admission) of the right corneal neoplasm showed small blood vessels on the surface of the lesion, as well as edema in the surrounding cornea. **d** Microscopic examination at the end of the operation showed clear incision after partial removal of the corneal neoplasm; residual neoplasm is also displayed in the image. **e** Preoperative color Doppler ultrasonography of the right eye showed protruded corneal neoplasm (white arrow) connected with the cornea and the iris. **f** Color Doppler ultrasonography at 10 months post operation showed decreased size of the neoplasm, smoother surface, and connection of the lesion with the cornea and the iris. **g** Bilateral eyes at 12 months post operation showed a white scar in the center of the right cornea. **h** T2W MRI at 12 months post operation showed a crumb-shaped neoplasm (white arrow) in front of the crystalline lens (black arrow), which was connected with the cornea; the iris and the ciliary body could not be clearly distinguished

A 4-month-old female infant presented to the Strabismus and Pediatric Ophthalmology Center in Tianjin Medical University Eye Hospital with sudden burst of a pink neoplasm in the right cornea and blepharospasm for one day. Previously, the patient had already been diagnosed with corneal dermoid in another hospital on postnatal day 7. She could fix and follow small toys with her left eye while patched the right eye, but cried loudly when the left eye was covered. A brownish-black hemispherical neoplasm (diameter, 4.5 mm) was observed in the center of the right cornea (Fig. 1a) with surrounding stromatic edema and extremely flat anterior chamber. In addition, a mild ciliary injection was observed in the right eye, with a much larger cornea (diameter, 12.0 mm). The anterior synechia of the iris was obviously beneath the lesion of the cornea, while the other internal structures of the right eye were not clearly visualized. The cornea of the left eye (diameter, 11.0 mm) was clear and no abnormality was found. Color Doppler ultrasonography was performed for the right eye under sedation, which revealed that the eyeball was intact, while a crumby lesion that linked the cornea and the iris was observed in front of the crystalline lens; however, the vitreous cavity was clear and no abnormality was found in the posterior segment (Fig. 1e).

The infant was the second child delivered normally by the mother at full term. General physical examination of the infant revealed no other noticeable abnormality. However, the mother had a history of repeated upper respiratory tract infection during the pregnancy.

Tobramycin eye drops were used four times a day to prevent infection in the right eye of the infant after admission. The corneal neoplasm in the right eye turned red on the next day (Fig. 1b); however, the volume of the lesion was apparently unchanged. The infant calmed down, and the symptoms of eyelid irritation disappeared. Initially, the baby was suspected to be suffering from corneal perforation, Peters’ abnormality, and congenital glaucoma. A surgical exploration on the right eye was performed under general anesthesia on day 4 after admission. During the operation, a red, firm, solid mass with dilated small vessels was found on the surface (Fig. 1c). No weakening or perforation was observed in the cornea. The protuberant part of the mass was excised, revealing the wound with a boundary that clearly differentiated it from the surrounding corneal tissues. The residual mass had a spiral-shaped presentation, while no distinct pigment tissue was found (Fig. 1d). Anterior chamber paracentesis was performed at 11 o'clock position of the limbus, and only a little aqueous fluid flowed out. Considering the possibility of pupillary block caused by anterior synechia, peripheral iridotomy was also performed. TobraDex eye ointment was applied to the eye, followed by eye patching. The mass was sent for pathological examination postoperatively.

**Fig. 2:**
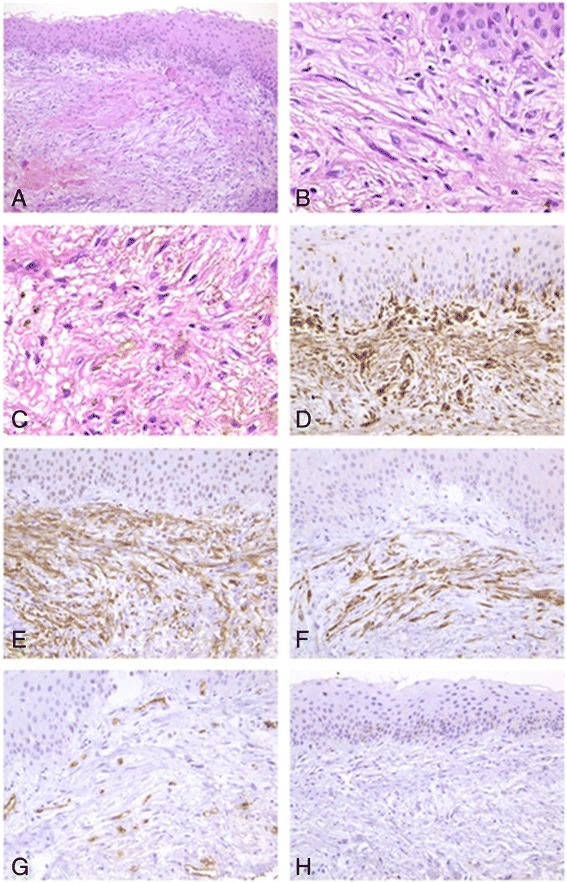
Results of hematoxylin & eosin (HE) and immunohistochemical staining of the neoplasm in the right cornea. **a** Regularly arranged multilayer squamous epithelial cells are seen on the surface of the neoplasm; no keratinization or atypia is observed. Numerous fibroblast-like cells are seen proliferated subepithelially, with a small amount of well-differentiated mature collagen fibers among them (HE × 100). **b** Microscopic examination under high magnification shows long, spindle- or bean-shaped cells, with abundant cytoplasm, arranged in bundles (HE × 400). **c** Brown pigment particles are seen among the tumor cells (HE × 400). **d** Tumor cells showing a high expression of vimentin (EnVision × 200). **e** Tumor cells showing SMA expression (EnVision × 200). **f** A proportion of the tumor cells expressing desmin (EnVision × 200). **g** The vascular endothelial cells among the tumor cells show CD34 expression (EnVision × 200). **h** No S100p expression is seen in the tumor cells (EnVision × 200)

Conventional hematoxylin & eosin staining revealed multilayers of well-differentiated mature squamous epithelia on the surface of the mass; however, the cells on basal layer were well arranged, without atypia. Numerous fibroblast-like cells were observed, with a small amount of mature collagen fibers and blood vessels (Figs. 2a and b). The tumor cells were spindle-shaped, and arranged irregularly. These cells were eosinophilic and full of cytoplasm, and the nuclei were oval or fusiform, and lightly dyed without obvious atypia or mitosis. Some cells showed slender cytoplasmic protuberances that connected with collagen fibers. In addition, scattered brown pigment particles were observed among the tumor cells. Immunohistochemical staining showed strong expression of vimentin and smooth muscle actin (SMA) in the tumor cells, while desmin was only partially expressed; however, no sign of S-100 protein or CD34 expression was found in these cells. In contrast, for vascular endothelial cells in the tumor tissues, CD34 was found to be positively expressed (Figs. 2c–g). Therefore, the pathological diagnosis for the lesion was myofibroblastoma in the cornea of the right eye. The parents were well informed and the baby was taken back to have a check every 3 months. At the 12-month follow-up, a scar was found in the cornea of the right eye, while the diameter of the cornea was not increased, and the intraocular pressure was normal (Fig. 1f). Color Doppler ultrasonography and magnetic resonance imaging (MRI) were performed for the right eye, which showed that the mass was restricted but connected with the cornea and the iris; the back boundary of the mass was in front of the crystalline lens, and no growth of the mass was observed (Figs. 1f and h).

## Discussion

Myofibroblastoma is a benign mesenchymal neoplasm composed of spindle-shaped cells in clusters and fascicles, as well as interspersed bands of hyalinized collagen. Myofibroblastoma was initially reported in the breast of a male patient by Tokeret et al. in 1981, and since then, it has also been identified in other parts of the body [1–3]. Histologically, myofibroblastoma consists of proliferated spindle cells that are arranged irregularly or in bunches, with relatively bulky collagen fibers among them. These spindle-shaped cells are mainly eosinophilic, with moderate amount of cytoplasm and fusiform nuclei. Immunohistochemical staining revealed that the tumor cells were differentiated into fibroblastic and myofibroblastic, which are strongly positive for vimentin and SMA, and weakly positive for desmin and CD34 [4–6].

Ocular myofibroblastoma is a very rare condition, and to the best of our knowledge, no case in children or infants has yet been reported. Recently, Costin et al. [7] reported a case of myofibroblastoma in an adult female, with orbital and intraocular involvement. The infant in the present case had an intraocular myofibroblastoma since birth. Doppler ultrasonography on admission showed that the tumor was located in front of the crystalline lens, protruded from the cornea, and connected with the iris tissues, while the structures of the retina and vitreous body were still clear and unaffected. Histopathological examination of the partially excised lesions revealed numerous long, spindle- or bean-shaped tumor cells that proliferated subepithelially, with small amounts of collagen fibers and blood vessels. The nuclei were oval or fusiform and no obvious atypia was observed. Immunohistochemical staining showed that vimentin and SMA were positively expressed in these tumor cells, while desmin was only partially expressed; in contrast, no sign of S-100 or CD34 protein expression was found in these spindle-shaped tumor cells. Histopathological and immunohistochemical examinations revealed that this tumor satisfied the pathological diagnostic criteria of myofibroblastoma. Brown pigment particles were also found scattered among the tumor cells, suggesting that the corneal mass had the same differential origin as the iris.

The corneal mass in this infant was observed immediately after birth; its initial presentation was a pink raised uplifted neoplasm in the central cornea, which was similar to a corneal dermoid. Epibulbar dermoid is congenital overgrowth of collagenous connective tissue covered by epidermoid epithelium in an abnormal location, including bulbar conjunctiva, limbus, cornea, and/or caruncles. Both the central cornea and the peripheral cornea may be involved. Occasionally hair follicles, sebaceous and sweat glands, or adipose tissues could be found insides [8]. However, the tumor in this infant mainly consisted of large amounts of spindle-shaped cells, while no cutaneous appendage was found. So that a corneal dermoid could be excluded.

The corneal mass of the infant ruptured and turned black before admission, which could have easily been misdiagnosed as corneal perforation. Spontaneous corneal perforation rarely occurs in infants. It could be associated with systemic diseases such as infectious endophthalmitis, keratitis, or Peter’s anomaly [9, 10]. The cornea of the infant in the present case showed no obvious ulcer, the vitreous cavity was clear, and the infant was healthy without obvious inflammatory diseases. Therefore, corneal perforation caused by endophthalmitis or keratitis should not be considered. To the best of our knowledge, thus far, only 4 cases of spontaneous corneal perforation accompanied by Peter’s anomaly in infants have been reported [11]. Peter’s anomaly is characterized by the absence of posterior corneal stroma and Descemet’s membrane with iris and/or crystalline lens adhesions that are induced by genetic or environmental factors. Opacity in the central or peripheral cornea usually exists at birth; most of the patients have a flat anterior chamber, and some of the cases, Peter’s anomaly could also be accompanied by glaucoma [12]. In the present case, iridocorneal adhesion was apparent in the affected eye of the infant, the corneal lesion was black and hemisphere-shaped, and the infant’s symptoms included lacrimation, photophobia, and blepharospasm. Hence, this condition could also have been easily misdiagnosed as Peter’s anomaly accompanied by corneal perforation. However, the image of color Doppler ultrasonography and MRI demonstrated a apparent mass inside the anterior chamber, which give a hint to differentiate from Peter’s anomaly either.

The clinical presentations of the condition in the present case and the results of color Doppler ultrasonography and MRI showed that the tumor was closely associated with the iris, suggesting that they may have had the same origin in the embryonic stage. The progressive forward growth of the mass eventually affected the differentiation of normal corneal tissues, caused a defect of the central corneal stroma, and finally resulted in a dome-shaped mass on the cornea. We also observed several brown pigment particles scattered among the tumor cells, which also strongly indicated that the corneal tumor could have originated from the iris. As the diameter of the affected cornea was much larger than that of the fellow eye, and since the central anterior synechia was also significant, secondary glaucoma due to pupillary block should have been considered. Peripheral iridectomy was performed to drain the aqueous fluid to the anterior chamber. At the 12-month follow-up, no increase in the corneal diameter of the affected eye was observed, and the intraocular pressure was normal. In addition, color Doppler ultrasonography was performed 3 times, which further confirmed that the lesion was well restricted and that the tumor showed no evident growth.

On the other hand, considering visual rehabilitation of the affected eye, penetrating keratoplasty may be hopefully effective if the tumor keeps stable with the time going. However, corneal transplantation in children is extremely complicated and there is a high risk of graft failures in the younger age group due to the lower rigidity of sclera and cornea, much severe inflammation and more active immune system [13–15]. Furthermore, thoroughly removal of the tumor may even accompany lensectomy or vitrectomy. These additional surgeries might augment the risk for irreversible graft rejection either [13]. Considering all above, we still keep on following to observe the further changes of this rare intraocular tumor and wait for the optimum time to perform operations for improvement of vision.

## Conclusion

Ocular myofibroblastoma is very rare up to our knowledge, especially in infancy. This case was involving anterior chamber and complicated with secondary glaucoma. Its manifestation should be differentiated from corneal dermoid and Peter’s anomaly. The HE and Immunohistochemical staining as well as the images of Color Doppler ultrasonography were vital to give a precise diagnosis.

## Consent

Written informed consent was obtained from the parents of the patient for publication of this case report and any accompanying images. A copy of the written consent is available for review by the Editor of this journal.
